# The Use of Functional MRI to Study Appetite Control in the CNS

**DOI:** 10.1155/2012/764017

**Published:** 2012-05-08

**Authors:** Akila De Silva, Victoria Salem, Paul M. Matthews, Waljit S. Dhillo

**Affiliations:** ^1^Section of Investigative Medicine, Division of Diabetes, Endocrinology and Metabolism, Imperial College London, Hammersmith Hospital Campus, Du Cane Road, London W12 0NN, UK; ^2^GlaxoSmithKline Global Imaging Unit, Imperial College London, Hammersmith Hospital Campus, Du Cane Road, London W12 0NN, UK; ^3^Centre for Neuroscience, Department of Medicine, Imperial College London, Hammersmith Hospital Campus, Du Cane Road, London W12 0NN, UK

## Abstract

Functional magnetic resonance imaging (fMRI) has provided the opportunity to safely investigate the workings of the human brain. This paper focuses on its use in the field of human appetitive behaviour and its impact in obesity research. In the present absence of any safe or effective centrally acting appetite suppressants, a better understanding of how appetite is controlled is vital for the development of new antiobesity pharmacotherapies. Early functional imaging techniques revealed an attenuation of brain reward area activity in response to visual food stimuli when humans are fed—in other words, the physiological state of hunger somehow increases the appeal value of food. Later studies have investigated the action of appetite modulating hormones on the fMRI signal, showing how the attenuation of brain reward region activity that follows feeding can be recreated in the fasted state by the administration of anorectic gut hormones. Furthermore, differences in brain activity between obese and lean individuals have provided clues about the possible aetiology of overeating. The hypothalamus acts as a central gateway modulating homeostatic and nonhomeostatic drives to eat. As fMRI techniques constantly improve, functional data regarding the role of this small but hugely important structure in appetite control is emerging.

## 1. Introduction

When energy intake exceeds energy expenditure in the long term, the excess accumulates as body fat. It is predicted that, by 2015, as many as 30% of adults in Europe will be obese, as defined by a body mass index (BMI) ≥30 kg/m^2^ [1]. It is well known that obesity confers increased risk to a range of diseases, including type 2 diabetes, cardiovascular disease, and cancer. An enhanced understanding of the physiological mechanisms that regulate appetite is essential for the development of pharmacological strategies to combat obesity. In particular, within the field of neuroendocrinology, identification of hormone-brain interactions that modulate appetite (and subsequent food intake) will aid the manipulation of naturally occurring hormones to develop safe and effective antiobesity therapies. This strategy, exploiting physiological pathways of appetite control, holds promise for the future treatment of obesity, especially given the shortage of effective pharmacotherapies for obesity in the existing market. For a comprehensive overview of the position of gut hormones in the treatment of obesity, we direct the reader to a review by Valentino et al., 2010 [2]. 

The control of appetite and food intake can be divided into “homeostatic” and “nonhomeostatic” control. The alteration in consumption of food that follows sensing of energy balance forms the basis of the homeostatic control of appetite: following a meal, appetite is suppressed, whereas, following significant energy expenditure, we feel hungry. Postprandial satiety signals include changes in circulating concentrations of nutrients and gut hormones. These include the anorectic hormones PYY and GLP-1 and the orexigenic hormone ghrelin [3], as well as activation of somatosensory vagal afferents conveying information such as gastric distension [4]. Adipokines (hormones secreted by adipose tissue, such as leptin) convey information to the CNS about longer-term energy stores [5]. Furthermore, it is well established that insulin is secreted acutely from the pancreas in response to a glucose load but is also basally elevated in states of overnutrition and obesity, thereby implying a role in conveying both short- and longer-term information regarding energy balance. In normal physiology, the integration of the above inputs within central neural appetite circuits results in appropriately matching food intake to metabolic need. The hypothalamus is widely recognised as the gatekeeper for this processing task, in line with other important homeostatic functions controlling body temperature, energy expenditure, and glucose metabolism. In keeping with this, there is mounting evidence that hypothalamic dysfunction is implicated in the pathogenesis of obesity [6].

**Figure 1:**
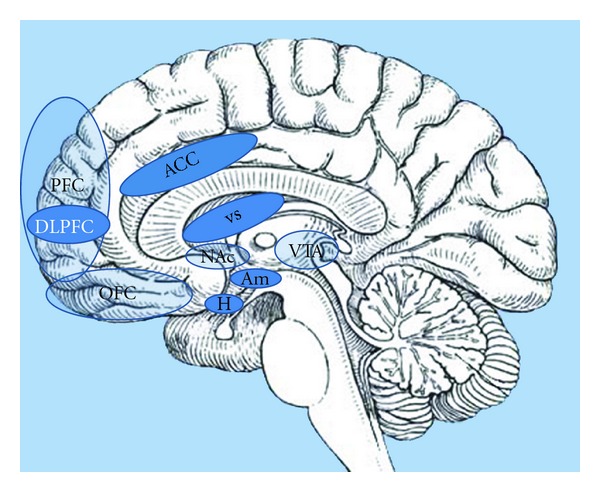
Brain reward centres: the hypothalamus (H), as a homeostatic gatekeeper, has numerous connections with higher brain centres which process salience and reward. The hypothalamus transmits to these higher centres information received from the periphery, such as nutritional status signalled via the postprandial release of gut hormones, and in turn modulates metabolic rate via the sympathetic nervous system. This sagittal section of the brain reveals the important areas involved in the hedonic control of eating behaviour; amygdale (Am): emotional and aversive processing; nucleus accumbens (Nac): anticipatory reward processing; ventral tegmental area (VTA): numerous dopaminergic projections to other limbic areas; ventral striatum (VS): motivation reward; expectancy and novelty processing; anterior cingulate cortex (ACC): decision making; orbitofrontal cortex (OFC): reward encoding; prefrontal cortex (PFC): translation of external and internal cues into behavioural responses; dorsolateral prefrontal cortex (DLPFC): self-control. Not shown is the insular cortex (a more lateral structure), which is also important in gustatory processing.

With regard to “nonhomeostatic” control, food intake is driven by factors including food palatability (incorporating the key senses of sight, smell, and taste), habitual, socio-cultural, emotional, and economic influences. From an emotional perspective, the evaluation of a food stimulus for its motivational significance occurs in multiple brain regions, particularly the dopaminergic limbic and prefrontal reward areas [7]. Key areas include the amygdala, hippocampus, insula, striatum, and orbitofrontal cortex (OFC), although this list is by no means exhaustive. It is now widely accepted that, with regard to appetite control, homeostatic and nonhomeostatic systems do not function independently; instead, there is extensive cross-modulation between them, along with a complex integration of inputs before a final decision is made regarding food consumption (Figure 1).

Over the past decade, functional magnetic resonance imaging (fMRI) has become a popular and rapidly advancing tool for investigating CNS appetite pathways in humans, offering the key advantage of avoiding exposure to ionising radiation. Following on from previous similar articles [8–10], here we provide an updated review of recent advances in our understanding of human appetitive behaviour based on the results of fMRI studies. We start with a brief overview of the technique and then summarise findings from the earliest fMRI appetite studies, which first explored differences in brain activation patterns between the normal fed and fasted state and in response to glucose ingestion. This is followed by a discussion of a further cluster of studies, exploring differences between obese and lean populations. Finally, we review the results of fMRI studies investigating hormonal influences on appetitive brain processes and in relation to this and explore recent functional neuroimaging outcomes following intervention in obese subjects.

## 2. Principles of fMRI in Appetite Studies

A full description of fMRI methodology is clearly outside the remit of this paper. It is nevertheless useful for the nonimaging specialist to understand some of the technicalities of fMRI, in order to gain a better appreciation of how the field has progressed, as well as some of its limitations.

**Figure 2:**
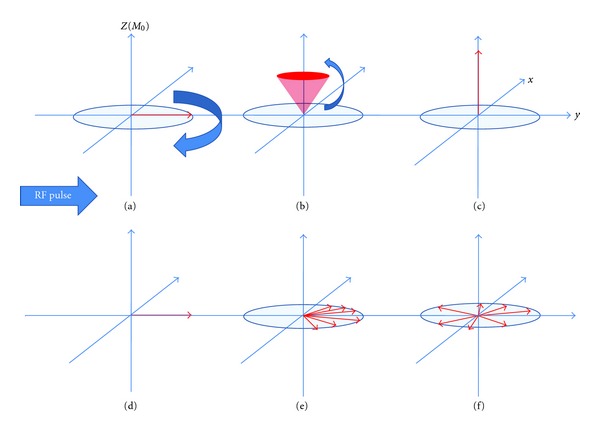
Schematic of *T*_1_ and *T*_2_ relaxation. MRI utilises the behaviour of protons within varying magnetic fields to produce signals which can be converted into images. Each hydrogen nucleus in the brain can be thought of as a vector (in the *z* and *x*-*y* planes) representing the strength and direction of its magnetic field as it spins on its axis (its magnetic dipole moment, MDM). The MDMs of the imaged protons try to align with the main external magnetic field of the scanner (referred to here as *B*_0_ and conventionally shown along the *z* axis in 3D coordinates). A second magnetic field (in the form of a short radiofrequency RF pulse) is applied, which flips all of the MDMs from alignment in the *z* direction into the *x*-*y* plane (a). Before application of the RF pulse the, amplitude in the *z*-axis is maximal while the amplitude in the *x*-*y* plane is zero. Just after application of the RF pulse the, amplitude in the *z*-axis is zero (a) while the amplitude in the *x*-*y* plane is maximal (d). During relaxation, the amplitude in the *z*-axis will slowly increase ((b) and (d)) while the amplitude in the *x*-*y* plane slowly decreases ((e) and (f)).  *T*_1_ relaxation is the time taken for the *z* vector to regain in strength, whereas *T*_2_ relaxation is the time taken for the *x*-*y* vector to decay. These changing magnetic vectors invoke their own RF signals, which are picked up by the receiver coils and interpreted into information about the proton density of the subject being scanned.

MRI utilises the behaviour of hydrogen nuclei, which consist of single protons that possess angular momentum (spin). As soon as an external magnetic field (*B*_0_) is applied, the protons in tissue tend to align with this, causing their spins to precess about a circular path around *B*_0_. Here, they are in a low-energy state. Generation of MRI images requires application of a radiofrequency (RF) pulse at 90 degrees to *B*_0_. The protons will then “tip” to align with the RF pulse. In doing so, they gain energy. After the RF pulse is switched off, the protons realign with *B*_0_. Spins return to the low-energy state by emitting the absorbed energy, also in the form of a radio wave. The emitted energy can be measured by the receiver coil and converted to images. By slightly altering the strength of the magnetic field (and therefore the frequency of the emitted radiation) using gradient coils across the volume to be imaged, spatial information can be inferred. The *T*_1_ relaxation time is a time constant referring to the realignment of spins with *B*_0_ in the longitudinal plane after the RF pulse is switched off. The *T*_2_ relaxation time, on the other hand, is a time constant referring to the dephasing of spins in the transverse plane after the RF pulse is switched off (Figure 2). *T*_1_ and *T*_2_ vary depending on the tissue being imaged. The strength of magnetic resonance signal obtained for a particular tissue depends primarily on the proton density. However, by altering the time between successive RF pulses and therefore the degree of *T*_1_ and *T*_2_ relaxation, the image can be weighted towards one or the other of these tissue-specific properties. It should be noted that, in practice, due to localised inhomogeneities in the externally applied magnetic field, *T*_2_ is shorter than expected for any particular tissue. This apparent *T*_2_ is referred to as *T*_2_*.

fMRI utilises adaptations of this classical MRI technique, such that function of tissues can be analysed rather than structure alone. Virtually, all fMRI studies rely on a measure called blood-oxygenation-level-dependent (BOLD) contrast, based on the fact that oxygenated and deoxygenated blood possess different magnetic properties. Increased neuronal activity in the brain elicits a local haemodynamic response, which causes an increase in blood flow greater than necessary for tissue demands. This results in a locally reduced ratio of deoxyhaemoglobin to oxyhaemoglobin concentrations, and the ensuing differences in local magnetic field inhomogeneities can be detected on a *T*_2_*-weighted imaging protocol [11, 12]; the locally reduced ratio of deoxyhaemoglobin to oxyhaemoglobin leads to a longer *T*_2_*, producing an increased image intensity. Such protocols typically use magnetic gradients to generate the MR signal (gradient-echo imaging) and, as scanner and computational hardwares have advanced, faster sequences have been developed to acquire large numbers of images in short spaces of time. Echo-planar imaging (EPI), for example, is a technique whereby an entire 2-dimensional image can be attained by the rapid alteration of spatial gradients following a single RF pulse. In this way, the entire brain can now be functionally imaged within the same timeframe as the physiological changes of interest. Functional MRI has thus provided hugely important insights in numerous disciplines. However, its use in the field of neuroendocrinology has been limited due to the inherent weakness of the technique at imaging the hypothalamus. This small (approximately 5–8 mm in diameter) brain structure lies close to the air-tissue boundaries of surrounding sinuses and is susceptible to signal loss due to resultant distortions of the external magnetic field, which is a particular issue for *T*_2_*-weighted images as already described [13]. 

fMRI image reconstruction results in the selected brain area divided into thousands of voxels, each assigned a signal intensity. Statistical analysis of each voxel or cluster of voxels ascertains whether the signal intensity there is greater than compared with all others. Whilst this approach may be useful for exploratory studies, most fMRI experiments perform ROI (region of interest) analyses whereby the functional properties of a predetermined set of voxels (corresponding to *a priori* anatomical areas of the brain) are analysed. ROI analysis, with very many fewer degrees of freedom than whole-brain voxelwise analysis, has a greater chance of finding statistically significant results but entails the inherent risk of missing activated areas of the brain that were not included in the original hypothesis. Resting state functional connectivity analyses investigate the activational interrelationships between different brain regions, allowing for the formation of distinct neuronal networks implicated in a coordinated brain response [14]. Resting state scans are performed when the subject is not performing any purposeful task and have recently reported on blood flow to the hypothalamus as an ROI with or without infusion of anorectic gut hormones [15].

Another commonly used approach in the area of functional imaging of appetite is to present the subject with images of food (with further subdivision into appetising, bland, high calorie, or low calorie food) or nonfood items. A subtraction analysis is performed to see whether the difference in regional brain activation between viewing images of food or nonfood is altered in different physiological conditions (e.g., the fasted state, the fed state, or in the presence of exogenously administered anorectic hormones). Several studies have utilised this approach; these will be discussed in depth subsequently. Hypothalamic data remain an elusive goal with such whole brain task-based analyses; in addition to the aforementioned difficulties in hypothalamic imaging, activation of some nuclei within the hypothalamus promotes food intake, whereas other nuclei are appetite-inhibiting [16]. Therefore, using the hungry, fasted state as an example, the overall hypothalamic signal resulting from the activation of orexigenic nuclei and inhibition of anorexigenic nuclei may in fact be negligible, due to the above opposing signals cancelling each other out. In addition to developing stronger magnetic fields to enhance spatial resolution, newer perfusion imaging techniques, which are *T*_1_ weighted, may provide better hypothalamic data which is not so affected by signal loss due to magnetic susceptibility artefacts [17]. Arterial spin labelling (ASL) uses RF pulses to magnetically label protons in blood water molecules before they reach the tissue of interest which lies in the imaging plane from where data is acquired.

## 3. Imaging the Hypothalamic Response to Glucose Using Midsagittal Slice Selection

Early studies that have concentrated on hypothalamic signal change involved conventional *T*_2_*-weighted gradient-echo MRI pulse sequences to functionally scan a sagittal plane through the hypothalamus continuously, acquiring images before- and after- a given stimulus. By concentrating all image processing ability on this thin, central slice, greater attention can be devoted to a ROI corresponding to the hypothalamus, although clearly at the expense of imaging the rest of the brain. In this manner, Matsuda et al. demonstrated that oral glucose ingestion produced a profound transient reduction in hypothalamic BOLD fMRI signal in lean subjects [18]. They alluded to the hypothalamic effects being mediated by neurohumoral factors following glucose ingestion, since the hypothalamic signal change preceded any significant rise in blood glucose. 

In 2000, Liu et al. described a new technique called time clustering analysis (TCA), whereby the number of voxels reaching maximum signal intensity as a function of time was examined [19]. In this way, TCA records the time windows in which maximal brain responses occur and from which ROI analysis can be appropriately extracted. By scanning a 1 cm midsagittal slice, they were able to further concentrate resolution on the hypothalamus and found that significant hypothalamic deactivation occurred 10 minutes following glucose ingestion. They also showed that the degree to which the hypothalamic signal reduced postglucose ingestion negatively correlated with fasting plasma insulin levels.

Using functional MRI in a 1 cm midsagittal slice, Smeets and colleagues reported on resting state intrasubject hypothalamic response before and after ingestion of glucose. In their original experiment, they also found a dose-dependent decrease in hypothalamic BOLD fMRI signal in lean, healthy subjects shortly after ingesting glucose solution, which lasted for 30 minutes [20]. Using the same experimental paradigm, they went on to show that hypothalamic BOLD signal did not decrease following ingestion of an equally sweet (but calorie deficient) solution of aspartame, or ingestion of an equally calorific (but not sweet) solution of maltodextrose. They thereby inferred that hypothalamic activity was specifically glucose-sensitive and that a hypothalamic response required both sweet taste and energy content [21]. Interestingly, from a clinical perspective, they subsequently demonstrated that the reduction in hypothalamic BOLD signal following glucose ingestion was absent in patients with type 2 diabetes, theorising that inappropriate hypothalamic processing of nutrient availability may be involved in the aetiology of the disease [22]. Using the same hypothalamic BOLD imaging technique as these previous studies, this group has also alluded to the effects of gut hormones on hypothalamic activity following glucose ingestion. In healthy men, despite causing a threefold lower rise in blood glucose concentration than intravenous glucose administration, oral glucose ingestion led to a much more robust reduction hypothalamic BOLD signal [23]. As well as the effect of taste, it was postulated that hormones such as GLP-1, released in response to nutrients entering the gut, accounted for this greater hypothalamic response to oral rather than intravenous glucose.

However, in contrast to the above findings, a recent study by Purnell et al. in 2011 did not detect any signal change in the hypothalamus in response to either glucose or fructose infusion in 9 healthy, normal-weight subjects, despite focussing their image acquisition parameters to optimise hypothalamic signal capture by mid-sagittal *T*_1_ weighting [24]. The authors postulated that differential activation of excitatory and inhibitory nuclei within the hypothalamus may have cancelled out any overall signal change. This illustrates the ongoing limitations when attempting to visualise the human hypothalamus in fMRI studies.

## 4. fMRI Studies in Normal-Weight Subjects Comparing the Response to Food Cues in the Fasted and Fed States

Before moving into the arena of functional brain imaging studies to specifically determine the neuroendocrinology of appetite control, it is useful to look at earlier studies which examined differences in brain activity in hunger and satiety. Such differences may be attributed to a number of physiological differences between the fed and fasted state, such as changes in concentrations of gut-derived neuropeptides (PYY, GLP-1, ghrelin, CCK, and insulin to name but a few) and vagal afferents conveying information about gut distension. These early studies into appetitive behaviour have led to our understanding of how images of food can trigger the brain's reward system and how the motivational potency of this trigger is greater in the fasted state. On the whole, these studies complemented earlier work which utilised PET scanning to assess the effects of hunger and satiety on regional cerebral blood flow differences and which particularly implicated the OFC as a critical convergence zone for sensory information related to rewarding stimuli [25–28].

Most of these fMRI studies have been task-based subtraction analyses, looking at differences in regional brain activation between when viewing images of food or nonfood items. It is well understood that, whilst taste provides an immediate reward (or punishment) for consumed foods, the visual characteristics of food are quickly learned and become powerful secondary reinforcers, capable of influencing subsequent food-seeking behaviour. Thus, showing food images is a useful way of examining the appetitive reward circuitry.

In the first study of its kind in 2001, LaBar et al. reported on 9 healthy subjects, who underwent an initial whole-brain fMRI after an 8 hour fast, followed by another postmeal scan one hour later. A follow-up study was performed on 8 subjects who were fed a meal before the first of the two scans to rule out the potential of habituation effects in the first group. The subjects were presented with food and nonfood images during each scan and subtraction analysis was performed [29]. The authors' hypothesis that variation in the state of hunger would modulate the response of the amygdala and anatomically related corticolimbic structures was borne out in a ROI analysis, which revealed satiety-induced reduction in activity in the amygdala, parahippocampal gyrus, and fusiform gyrus. They suggested that the amygdala, with its extensive neural connections with the hypothalamus and higher brain centres, was in a pivotal position for integrating response to visual food stimuli in the context of nutritional status. However, it was noted that this study was underpowered to detect similar satiety-induced changes in many other ROIs studied.

In 2003, Killgore et al. used a different approach by scanning satiated recruits who were presented with pictures of high calorie, low calorie, and nonfood items. Irrespective of calorific content, food images caused greater activation than nonfood images in the amygdala, hippocampus, and ventromedial prefrontal cortex [30]. The authors point out the importance of these areas in the expectation and evaluation of reward. High-calorie foods caused particular activation of the medial and dorsolateral prefrontal cortex, understood to be involved in evaluating stimulus relevance within the current affective state of the individual. In contrast, low calorie foods resulted in lesser activation of these reward areas per se and greater activation in somatosensory areas, with a suggestion from the authors that this was due to a lesser cephalic phase response by classical conditioning to images of less appealing foods. In 2005, Killgore published a reanalysis of this study data, using an ROI approach (specifically, a subanalysis of the OFC) and correlated BOLD activity with BMI. It was reported that, for the high calorie minus nonfood contrast, there was a significant negative correlation between BMI and BOLD signal in the OFC [31]. In other words, as BMI increases, activity in the OFC becomes less food responsive. This highlights the more subtle role of parts of the OFC not just in simply assigning reward value to certain stimuli, but in modifying stimulus-reward associations and accordingly redirecting feeding-related behaviour in response to new learning [32]. Of course, in this particular study, it was not possible to extend these findings to the obese population, since the subjects studied were all within the normal weight range.

In 2005, St-Onge et al. published the only fMRI study to date where fasted individuals were exposed to four different stimuli: visual food, visual nonfood, tactile food, and tactile nonfood. In an uncorrected whole brain analysis, they reported that the anterior cingulate, superior temporal gyrus, hippocampus, and insula were significantly activated to a greater extent during the presentation of foods (whether seen or felt) over nonfood items [33].

**Figure 3:**
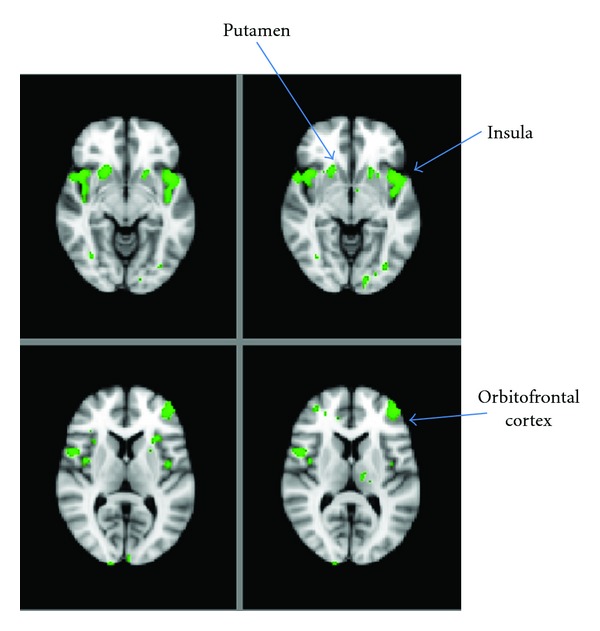
Modulation of neuronal activity in the fed versus fasted state. Representative whole-brain fMRI sections showing regions where the difference in BOLD signal between viewing food images and nonfood images is blunted in the fed state compared with the fasted state. Unpublished image from [37].

In 2006, Porubská et al. published the results of an fMRI study of 12 normal-weight fasted subjects. Visual food stimuli (in contrast to nonfood images) activated the insular and orbitofrontal cortices, with a positive modulation of insular activity induced by subjective ratings of appetite [34]. These findings are consistent with the recognition of the insula as being an important region in establishing salience [35]. Following on from this, Führer et al. in 2008 studied 12 healthy male volunteers undergoing two separate scanning sessions—one when fasted overnight and the other immediately after a large meal. They performed a whole brain, uncorrected analysis of the data and particularly noted significantly enhanced activity within the OFC when hungry, again with reference to our understanding of this area as subjectifying the perceived pleasantness of food [36]. We have also recently demonstrated that feeding reduces the difference in BOLD signal between viewing images of food and nonfood in several brain regions (Figure 3), but significantly so in the insula [37]. Furthermore, in 2009, Goldstone et al. reported the findings from a fMRI study of twenty individuals in both the fed and fasted state; subjects viewed pictures of high calorie, low calorie, and nonfood items whilst rating the appeal value of these images. They found that when fasted, there was significantly greater activation to high calorie over low calorie food items in the ventral striatum (important in mediating hedonic drive and action [38]), amygdala, anterior insula, and OFC. They found that high calorie foods were consistently rated as more appealing, that this was augmented with fasting and that the increase in appeal rating bias for high calorie over low calorie foods in the fasted state was positively correlated with activity in the OFC [39]. In the same year, Schur et al. performed a study of ten normal weight, fed subjects viewing images of fattening food, nonfattening food, and nonfood items. These food images were specifically chosen based on whether the food was perceived to be compatible with an effort to lose weight. In a ROI analysis, this was the first study to report increased hypothalamic activation when viewing pictures of fattening food compared with nonfood items, although this finding did not extend to other comparisons (i.e., fattening versus nonfattening or all food versus nonfood). In concordance with other studies, they also found increased activation in the amygdala, insula and OFC when viewing fattening foods compared with nonfood items [40]. Also in agreement with previous findings were the results of Siep et al., who in 2009 reported increased activity in the amygdala and OFC in response to viewing high calorie versus low calorie food images, but only when their subjects were fasted [41]. Their experimental protocol allowed for the further observation that this increased activity in the amygdala and OFC was only evident when participants explicitly evaluated foods but not when their concentration was diverted elsewhere.

## 5. Differing fMRI Responses in Obesity

Bearing in mind the results of the above studies in normal weight individuals, it is now interesting to turn to the results of similar studies comparing obese and normal weight subjects.

As discussed above, in 1999, Matsuda et al. studied the hypothalamic response before and after oral glucose ingestion. Although they showed that oral glucose ingestion produced a profound transient reduction in hypothalamic signal in 10 lean subjects, this response was both significantly delayed and attenuated in 10 obese individuals. More tellingly, it was shown that, following glucose ingestion, the time taken to reach the maximum hypothalamic inhibitory response was closely correlated to fasting plasma glucose and insulin levels (which, as expected, were significantly higher in the obese group) rather than BMI per se, suggesting hypothalamic dysfunction as either the cause or consequence of insulin resistance [18].

To extend the above findings to the study of reward regions, a later study incorporating visual food and nonfood cue methodology revealed that BOLD fMRI activation in the right hippocampus (with respect to high calorie food images versus other images) correlated positively with fasting insulin levels and waist circumference (but not BMI) in 12 normal weight and 12 obese adolescents who had all been fed a standard breakfast a few hours prior to scanning [42]. On the other hand, in that study, there was significant negative correlation between activation of the medial right superior frontal gyrus and the left thalamus (again, with respect to the contrast between high calorie food images versus other images) with fasting insulin levels. The authors postulated that their results reflected a permissive role of insulin in the control of eating behaviour by the hippocampus. We further speculate that, in those with a larger waist circumference and high circulating levels of insulin (i.e., peripherally insulin resistant individuals), a degree of central insulin resistance in the hippocampus may explain the greater activation in this region, despite higher insulin levels.

Studies investigating the differential reward system activation patterns when looking at high calorie foods between obese and normal weight subjects have shown significantly greater activation in the obese group in several brain regions implicated in food reward. In a study by Rothemund et al. in 2007 of 13 obese and 13 normal weight women who had been fasted for at least 90 minutes, increasing BMI positively predicted BOLD activation of the dorsal striatum (caudate/putamen), anterior insula, claustrum, posterior cingulate, postcentral cortex, and lateral OFC. This specifically pertained to the contrast between viewing high calorie food images versus nonfood images [43]. A further ROI-based study by Stoeckel et al. in 12 obese and 12 normal weight women demonstrated that pictures of high calorie foods (versus nonfood) produced greater activation in the obese group compared with controls in several brain regions, including the ventral and dorsal striatum, insula, anterior cingulate cortex, amygdala, OFC, hippocampus, ventral pallidum, and medial prefrontal cortex [44]. A further functional connectivity analysis indicated that obese women displayed a relative deficiency in the amygdala's modulation of the OFC and ventral striatum, along with excessive modulation of the ventral striatum by the OFC [45]. Collectively, results from the above studies suggest that a hyperreactive reward system to high calorie food cues is involved in the pathophysiology of obesity.

Fascinatingly, several studies have shown that obese individuals respond differently to satiation compared with normal weight individuals, which allows us to hypothesize that, in obesity, there is a degree of dysregulation in CNS appetite regions, which may contribute to overeating and hence weight gain.

A ROI-based study by Martin et al. measured fMRI BOLD activation pre- and postprandially (following a 500 kCal meal) in response to pictures of food (a combination of high calorie and low calorie images) and nonfood in 10 obese and 10 healthy weight adults. During the premeal condition, obese subjects showed increased activation in the anterior cingulate and medial prefrontal cortex, compared to healthy weight controls [46], thus concurring with the observations of Stoeckel et al. During the postmeal condition, obese participants also showed greater BOLD activation in the medial prefrontal cortex compared with healthy weight controls. A further recent study by Dimitropoulos et al. of 22 overweight/obese males and 16 normal weight males showed that before eating, obese subjects showed greater response to food images (versus nonfood images) compared with normal weight subjects in the anterior prefrontal regions [47]. Postprandially, the obese group demonstrated increased response to all food images (versus nonfood) compared with normal weight individuals in frontal, temporal, and limbic regions. Specific greater activation to high calorie foods was seen in the obese group compared with normal weight individuals in the lateral OFC, caudate, and anterior cingulate cortex [47].

An interesting hypothesis to explain abnormal eating in obesity was postulated by Stice et al., following an fMRI study in 2008. Here, a comparison was made between brain activity in 7 obese and 11 lean adolescent girls during the anticipated receipt of chocolate milkshake, during actual receipt of the milkshake, during anticipated receipt of a tasteless control solution, and during actual receipt of the tasteless control solution. In response to anticipated receipt and actual receipt of the chocolate milkshake (versus the tasteless solution), obese adolescent girls showed greater BOLD activation bilaterally in the anterior and mid insula, frontal operculum, parietal operculum, and rolandic operculum compared with lean adolescent girls. However, the obese girls displayed lower BOLD activation in the caudate in response to actual consumption of the milkshake (versus the tasteless solution) compared with the lean girls. From these results, the authors suggested that obese individuals show greater salience-associated responses from anticipated food consumption in gustatory and somatosensory regions, but weaker activation in the caudate during actual consumption compared to lean individuals [48]. They further postulated that this reflects increased anticipatory food reward (but reduced consummatory food reward) in obesity, which may contribute to overeating. In a further study, after baseline characterisation, 8 women who showed >2.5% increase in BMI over a 6-month period showed reduced striatal BOLD fMRI response to chocolate milkshake consumption (versus a tasteless control solution) compared with 12 women who demonstrated stable weight [49]. These results reinforced the authors' hypothesis that weight gain may be associated with reduced sensitivity of striatal reward circuitry (probably due to the downregulation of dopamine D2 receptors), which may be a fundamental mechanism responsible for overeating.

Differences between normal weight and obese individuals have been replicated in studies of children; in one such investigation of 10 obese and 10 normal weight children, in the fasted state, the obese group showed greater BOLD activation (when viewing food images versus nonfood images) in the prefrontal cortex compared with the normal weight group. Following a meal, the reduction in activation of the prefrontal cortex and nucleus accumbens was blunted in the obese group. Furthermore, the postmeal activation of the OFC (to food versus nonfood images) was greater in the obese group compared with the normal weight group [50].

## 6. fMRI Studies of Hormones Implicated in Appetite Control

Having discussed the first generation of fMRI studies exploring the physiological responses to feeding, followed by studies investigating alteration of such responses in obese individuals, we will now direct our focus to research that has investigated the effects of appetite modulating hormones derived from the gastrointestinal tract and adipose tissue on the fMRI neuronal response. As alluded to earlier, such hormones are thought to be key mediators of both short-term and longer-term energy balance; in the postprandial state, anorectic hormones (including PYY, GLP-1, and insulin) predominate, and in the longer-term, high-circulating levels of leptin and insulin correlate with nutritional status. In contrast, in the fasted, hungry state, the orexigenic hormone ghrelin is dominant amongst gut hormones. It is therefore interesting to observe the effects of exogenous administration of such hormones on the fMRI response and compare these results with outcomes from earlier studies comparing the physiological fasted and fed states. It is worth noting that, thus far, the majority of fMRI studies coupling the exogenous administration of appetite modulating hormones (with the exception of studies of leptin) have been conducted in healthy, normal weight subjects, in order to investigate the action of such hormones in normal physiology.

### 6.1. PYY and GLP-1

PYY and GLP-1 are anorectic hormones, released by the enteroendocrine L cells of the gut following a meal; they lead to marked inhibition of food intake when administered to fasted human subjects [51–53]. In healthy human subjects, following intravenous infusion of PYY_3−36_, there was not only reduced food intake, but increased activity in the hypothalamus and OFC, as assessed by BOLD fMRI [15]. This study was conducted with subjects at rest, without engagement in a visual food-cue-based task. Furthermore, during saline infusion visits, subjects' caloric intake correlated positively with BOLD signal change in the hypothalamus, whereas this switched to a negative correlation between caloric intake and OFC signal on study visits when PYY_3−36_ was infused. It was postulated that the presence of PYY_3−36_ switched regulation of food intake from a homeostatic brain region (hypothalamus) to a hedonic region (OFC).

Using a visual food-cue-activated BOLD fMRI method, we recently investigated the effects of PYY_3−36_ and GLP-1_7−36__amide_ on neuronal activity in brain regions contributing to appetitive processing and behaviour [37]. We scanned 15 healthy, lean subjects on five separate occasions, during which they each received the following interventions in randomised order after an overnight fast: a saline infusion; a saline infusion following a large breakfast; an infusion of PYY_3−36_; an infusion of GLP-1; a combined infusion of PYY_3−36_ + GLP-1. Combined infusion of the gut hormones reduced *ad libitum* energy intake at a subsequent buffet meal to a similar degree as on the day when subjects received breakfast without either gut hormone. Notably, the BOLD fMRI signal change (comparing exposure to food images versus nonfood images) in several *a priori* brain ROIs was reduced following consumption of breakfast (Figure 3) and also when the gut hormones were infused in the fasted state. The largest reduction in BOLD signal occurred in the insula with combined administration of PYY_3−36_ and GLP-1, although we detected smaller reductions following feeding and gut hormone administration (either singly or in combination) in the striatum and OFC. Of note, our image acquisition parameters were not designed to optimise hypothalamic capture in favour of reward regions. Furthermore, when comparing our results for the OFC with those of Batterham et al., it must be remembered that a direct comparison between studies is difficult due to the methodological differences between them. Nevertheless, our study concluded that combined administration of the anorectic gut hormones PYY_3−36_ and GLP-1 to fasted individuals was associated with brain activation and subsequent eating behaviour changes similar to those after eating a full meal, thereby reinforcing the view that PYY and GLP-1 are key mediators of postprandial satiety.

### 6.2. Ghrelin

Ghrelin is a potent orexigenic gut hormone released from the stomach in response to fasting; administration of ghrelin to humans increases food intake [54]. A study by Malik et al. demonstrated that in normal-weight human subjects, intravenous ghrelin infusion increased hunger ratings and also increased the BOLD fMRI signal change (between food and nonfood visual cues) compared with saline infusion in the amygdala, OFC, insula, visual areas, and striatum [55]. The fMRI methodology used in this study was similar to that used in our PYY and GLP-1 investigation, so it is interesting to compare the results; whereas the anorectic hormones PYY and GLP-1 reduced BOLD signal in reward regions, the action of the orexigenic hormone ghrelin did the reverse, presumably in keeping with the opposing physiological roles of these peptides.

### 6.3. Insulin

Insulin has a central anorectic effect in normal physiology [56]. This must be distinguished from the secondary effect of hunger resulting from hypoglycaemia that may follow the exogenous administration of insulin (the associated overcompensatory eating may provide some explanation as to why patients with diabetes treated with insulin tend to gain weight). Therefore, human studies investigating the direct physiological action of insulin in the CNS have had to overcome the hurdle of maintaining euglycaemia during insulin administration, for otherwise, it is the effect of hypoglycaemia (rather than the effect of insulin per se) that would manifest. Furthermore, to effectively study the action of insulin in the CNS, peripheral effects of the hormone should ideally be eliminated, hence even the use of a euglycaemic hyperinsulinaemic clamp technique is unsatisfactory in this regard. One group has used intranasal insulin administration as a method of overcoming this technical difficulty, demonstrating that insulin resulted in reduced BOLD fMRI activation (with respect to food images versus nonfood images) in 9 healthy, normal-weight adults compared with placebo in the right and left fusiform gyrus, the right hippocampus, right temporal superior cortex, and the right frontal middle cortex [57]. Notably, peripheral plasma glucose did not change following the intranasal insulin administration, thus validating their technique of observing the central effects of insulin.

### 6.4. Leptin

Leptin-deficient individuals are obese and hyperphagic; replacement of leptin in these individuals reduces food intake [58]. However, the well-recognised phenomenon of leptin resistance in obesity has precluded its development from a therapeutic viewpoint. fMRI studies of leptin administration are nevertheless interesting to shed further light on the physiological regulation of appetite.

In 2 congenitally leptin deficient human subjects, daily subcutaneous leptin replacement reduced BOLD fMRI activation (in terms of the difference in activation between viewing food and nonfood images) in the nucleus accumbens-caudate and putamen-globus pallidus regions compared with the control, leptin-deficient state. Furthermore, before leptin treatment, activation in the nucleus accumbens-caudate correlated positively with liking of food images in both the fed and fasted states, whereas, after leptin treatment, activation in the nucleus accumbens-caudate correlated positively with liking of food images only in the fasted state [59]. Another study that year demonstrated that, in 3 congenitally leptin deficient human subjects, daily subcutaneous leptin replacement reduced the difference in BOLD fMRI activation (between viewing high-calorie and low-calorie food images) in regions implicated in the insula, temporal and parietal cortex compared with when subjects were not receiving leptin. On the other hand, leptin replacement increased the difference in BOLD fMRI activation (between viewing high-calorie and low-calorie food images) in the prefrontal cortex compared with when subjects were not receiving leptin [60]. The authors postulated that the insula, temporal cortex, and parietal cortex are hunger regions and therefore are inhibited by leptin, whereas the prefrontal cortex is a satiety region and is thus inhibited by weight loss and activated by leptin.

Following this, a study by Rosenbaum et al. in 2008 showed that in 6 leptin replete obese human subjects who achieved an initial 10% weight loss (between 36 and 62 days) through dietary means, there was increased BOLD fMRI signal difference (between comparing food images and nonfood images) in several regions including the brainstem and parahippocampal gyrus compared with the baseline state before weight loss. These changes were reversed by 5 weeks of subcutaneous leptin administration. Interestingly, other regions, including the hypothalamus and cingulate gyrus responded in the opposite direction following weight loss, but these changes remained sensitive to reversal by 5 weeks of subcutaneous leptin administration [61]. This study reinforced the idea that weight loss leads to a state of relative leptin deficiency, which may be responsible for the rebound hyperphagia and subsequent weight gain in many dieters.

**Figure 4:**
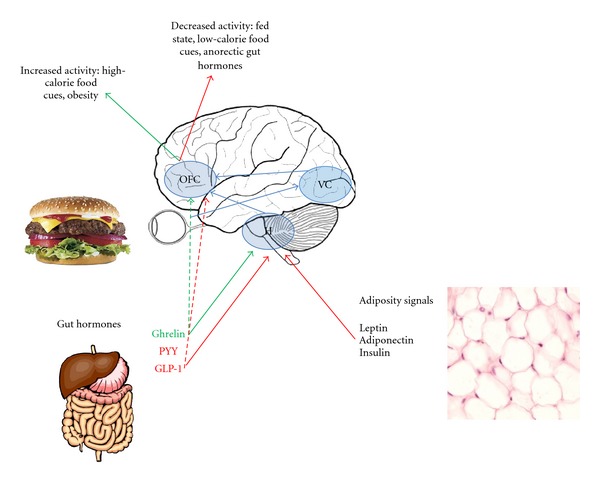
Modulation of the rewarding aspects of food. In this highly simplified schematic, the orbitofrontal cortex (OFC) is highlighted as the most important hub in the reward encoding network. External food cues, via the visual cortex (VC), modulate the OFC response, with increased activity seen in fasted and obese patients and in response to high- versus low-calorie foods. OFC activity is also thought to be modulated by inputs from the hypothalamus, which senses internal information about nutritional status in the form of adiposity signals (such as leptin, which gives information about longer-term energy stores) and gut hormones (which are meal dependent and therefore give information about shorter-term nutrient availability). Anorectic (postprandial) gut hormones, such as PYY and GLP-1, attenuate OFC activity and, in fasted individuals induce, an OFC response to visual food cues more similar to that measured when fed. Conversely, the orexigenic hormone ghrelin upregulates reward centre activity.

Figure 4 shows a simplified schematic of the modulation of appetite centres by peripheral signals via the hypothalamus and in obesity.

## 7. Imaging before and after Gastric Bypass Surgery

The most effective treatment for obesity available at present is Roux-en-Y gastric bypass surgery, leading to sustained weight loss of approximately 30% and often a very prompt resolution of type 2 diabetes [62].

Although the mechanisms behind the weight loss seen following Roux-en-Y bypass surgery remain to be fully elucidated, they are unlikely to be solely due to restrictive or malabsorptive means alone. Roux-en-Y results in greater weight loss than adjustable gastric banding, and this is thought to be due to a number of additional neurohormonal causes [63]. These include elevated postoperative levels of endogenous anorectic gut hormones such as PYY and GLP-1 [64–66], increases in energy expenditure and metabolic rate [67], changes in taste preference away from calorie dense foods [68, 69], increased bile acid delivery to the ileum [70], and changes in gut microbiota [71]. Recent studies imaging appetite in patients before and after weight loss are important in helping us to understand some of the CNS-mediated effects.

In 2011, Van De Sande-Lee performed fMRI studies on 8 lean control subjects and 13 obese patients before- and after- Roux-en-Y gastric bypass, by which an average 30% reduction in body weight had been achieved [72]. They replicated the TCA experiment described earlier [19]. In addition, they also performed a functional connectivity analysis. Their TCA findings mirrored that found previously—namely that the reduction in hypothalamic signal seen 5–10 minutes following glucose ingestion in normal controls was diminished in the obese (preoperative) group. However, a recovery of this impaired hypothalamic response was seen postoperatively when the obese group had lost weight following gastric bypass surgery. Interestingly, they also reported, in lean subjects, a high level of functional connectivity between the hypothalamus and the orbitofrontal and somatosensory cortices. This was not seen in obese subjects, but postoperative weight loss was reported to re-establish these connectivity patterns [72].

In the same year, a further ROI-based study of 10 obese female patients 1 month before- and after- Roux-en-Y gastric bypass, there was attenuation of fMRI BOLD signal (when viewing high-calorie versus low-calorie food images) in the ventral tegmental area, ventral striatum, putamen, lentiform nucleus, posterior cingulated, and dorsomedial prefrontal cortex following the bypass procedure [73]. In this study, hunger was also reduced after surgery. Although circulating gut hormone levels were not measured, the authors speculated that postsurgical reductions in the orexigenic hormone ghrelin could have explained their imaging observations. This would certainly be consistent with the previous fMRI findings by Malik et al. [55] following exogenous ghrelin administration. Equally, it is possible that elevated levels of PYY and GLP-1 after-bypass would explain the imaging findings, consistent with our recent fMRI findings [37] following exogenous administration of these anorectic hormones. Indeed, the elevation in PYY and GLP-1 levels achieved in our study following exogenous administration of the hormones was similar to the elevated endogenous levels of PYY and GLP-1 observed in previous studies following gastric bypass surgery [65]. These findings are striking, given the need to develop nonsurgical solutions for the treatment of obesity. To date, all centrally acting drugs developed to treat obesity have failed due to cardiovascular and mood altering side effects, as a result of actions outside pathways solely governing appetite. Therefore, it is foreseeable that, in the future, elevating circulating levels of PYY and GLP-1 (by means of exogenous administration) to similar levels as found naturally after a gastric bypass procedure may represent a novel pharmacological method of treating obesity.

## 8. Conclusion

The use of fMRI to study appetite control in humans is a rapidly evolving field. There appears to be a degree of consistency between studies, suggesting that, in the fasted state, greater BOLD activation is seen in several brain regions implicated in reward processing when food cues (and in particular high calorie food cues) are presented to subjects. Obese subjects seem to show heightened response to such food cues, which may fundamentally contribute to increased wanting of food (and thus increased subsequent food intake) in obese individuals. The fMRI data also seems to be in agreement that the consumption of a meal (or other surrogate markers of feeding, such as a rise in glucose) tends to deactivate key appetite regions in normal weight individuals and, furthermore, that this satiety response may also be abnormal in those with obesity. Further studies should lead to consolidation of existing concepts on the role of individual brain regions and their interconnections in physiological and pathological satiety responses.

Building on this fundamental understanding of the change in BOLD signal between the fasted and fed states, recent studies have suggested that, in general, orexigenic signals, which are in abundance in the fasted state, increase the BOLD response in brain reward regions, whereas anorectic signals, which predominate in the fed state, correspondingly deactivate the same regions. Subtle differences in brain responses between these studies may reflect the slightly different roles of the different hormonal signals in short-term versus longer-tem energy homeostasis and also may reflect differential action on various appetite pathways. Nevertheless, studies in obese patients, assessing the effect of gastric bypass surgery on the fMRI BOLD response, have further concurred with the results of studies investigating the BOLD response following the exogenous administration of orexigenic and anorectic hormones, thus consolidating the overall body of work in the area.

Although fMRI is at present used only as a research tool to study appetite, the overall goal in the future will be to translate this research into the clinical arena. An obvious role would be to help develop pharmacological treatments for obesity, by means of assessing the fMRI response to administration of novel agents in obese individuals. Alternatively, we may envisage the use of fMRI as a tool to help predict response to gastric bypass surgery. To aid effective comparison of results, it would be enormously useful to strive towards greater consistency between studies, particularly with regard to experimental design, image acquisition and image analysis. The studies outlined in this paper have established key concepts, and it is expected that the coming decade should see consolidation of these findings. We are fortunate that functional MRI studies have allowed scientists to investigate for the first time in humans, the intricacies of appetitive processing. As this technology continues to evolve, it will remain an important tool for investigating basic physiology as well as evaluating new therapies.
